# Plant Bioelectrical Signals for Environmental and Emotional State Classification

**DOI:** 10.3390/bios15110744

**Published:** 2025-11-05

**Authors:** Peter A. Gloor

**Affiliations:** 1 System Design and Management, Massachusetts Institute of Technology, Cambridge, MA 02139, USA; pgloor@mit.edu; 2 Cologne Institute for Information Systems, University of Cologne, 50923 Cologne, Germany; 3 Galaxylabs.org, CH-5000 Aarau, Switzerland

**Keywords:** plant bioelectrical signals, environmental sensing, emotion detection, convolutional neural networks, XGBoost classification, tradescantia pallida, mel-spectrograms

## Abstract

In this study, we present a pilot investigation using a single Purple Heart plant (Tradescantia pallida) to explore whether bioelectrical signals for dual-purpose classification tasks: environmental state detection and human emotion recognition. Using an AD8232 ECG sensor at 400 Hz sampling rate, we recorded 3 s bioelectrical signal segments with 1 s overlap, converting them to mel-spectrograms for ResNet18 CNN (Convolutional Neural Network) classification. For lamp on/off detection, we achieved 85.4% accuracy with balanced precision (0.85–0.86) and recall (0.84–0.86) metrics across 2767 spectrogram samples. For human emotion classification, our system achieved optimal performance at 73% accuracy with 1 s lag, distinguishing between happy and sad emotional states across 1619 samples. These results should be viewed as preliminary and exploratory, demonstrating feasibility rather than definitive evidence of plant-based emotion sensing. Replication across plants, days, and experimental sites will be essential to establish robustness. The current study is limited by a single-plant setup, modest sample size, and reliance on human face-tracking labels, which together preclude strong claims about generalizability.

## 1. Introduction

Plants generate sophisticated electrical signals through membrane potential changes, ion channel dynamics, and intercellular communication networks that have evolved over millions of years to process environmental information. Recent advances in bioelectronics and machine learning create unprecedented opportunities to decode these biological signals for technological applications, representing a paradigm shift from traditional electronic sensors to bio-informational systems that leverage plants’ intrinsic sensing capabilities.

Plant electrophysiology encompasses three primary signal types: action potentials (APs) with characteristic self-propagating depolarizations, variation potentials (VPs) triggered by environmental stress, and system potentials (SPs) involving H^+^-ATPase activation [1,2]. These electrical signals propagate through phloem elements and plasmodesmata, encoding stimulus-specific information through frequency patterns, signal amplitude variations, and temporal dynamics. The resting membrane potential in plant cells typically ranges from −100 to −200 mV, maintained by sophisticated ion transport systems including Ca^2+^ channels, anion channels, and voltage-gated K^+^ channels [3,4].

Recent research has demonstrated plants’ sensitivity to human interactions through multiple modalities. Oezkaya and Gloor (2020) first demonstrated individual recognition and emotion detection using plants as biosensors through electrostatic discharge measurements, establishing foundational evidence for plant–human bioelectrical communication [5]. Subsequent investigations by Kruse et al. (2024) leveraged deep learning approaches to recognize human emotions through plant sensitivity, achieving significant classification performance that validates the feasibility of emotion detection through plant bioelectrical responses [6]. Gil et al. (2024) extended this research by demonstrating plants’ capacity to perceive human gestures through AI-enabled tracking of eurythmic human–plant interactions, suggesting sophisticated multi-modal sensing capabilities [7]. These advances position plant-based sensing within broader frameworks of sustainable AI systems, as demonstrated by Tabuenca et al. (2024) in their integration of biological sensors with Internet of Things architectures for environmentally conscious smart learning environments [8].

Environmental stimuli trigger distinctive electrical responses in plants through well-characterized mechanisms [9,10]. Light stimulation activates cryptochromes and phytochromes, generating characteristic depolarization–hyperpolarization patterns, while temperature changes induce Ca^2+^ waves through mechanosensitive channels. Touch stimulation produces receptor potential-like responses, and chemical stress causes concentration-dependent membrane potential changes. These multi-modal sensing capabilities position plants as sophisticated biological sensors capable of integrating complex environmental information.

The application of machine learning to bioelectrical signals has shown remarkable success in medical diagnostics, with convolutional neural networks (CNNs) achieving over 99% accuracy in ECG classification tasks [11]. Transfer learning from ImageNet-trained models enables effective feature extraction for time–frequency representations of biological signals, while mel-spectrograms provide perceptually motivated frequency scaling that emphasizes biologically relevant low-frequency components.

This study addresses a critical gap by systematically investigating the use of Purple Heart plant bioelectrical signals for dual classification tasks: environmental state detection through lamp on/off recognition and human emotion classification. Our research questions investigate whether plant bioelectrical patterns contain sufficient discriminative information for environmental sensing applications and explore the temporal dynamics of plant responses to human emotional states.

## 2. Related Work

### 2.1. Plant Electrophysiology and Environmental Sensing

Plant bioelectrical signaling research goes back to Burdon-Sanderson’s 1873 recordings of Venus flytrap action potentials [12], with modern understanding shaped by Volkov’s comprehensive electrophysiology studies [13] and Fromm and Lautner’s systematic investigation of signal propagation mechanisms [14]. Contemporary research demonstrates plants’ remarkable sensing capabilities; Hedrich et al. (2016) [15] identified calcium signatures matching membrane potential dynamics across diverse stimuli, while Sukhov et al. (2021) [16] established stimulus-specific encoding through frequency patterns and signal characteristics.

Commercial applications have emerged through companies like Vivent Biosignals, whose PhytlSigns system provides AI-enabled crop health diagnostics using plant electrical signals, achieving early pathogen detection before visible symptoms appear. Research demonstrates 99.29% accuracy in plant stress detection using machine learning models applied to electrophysiological signals [17], with applications spanning precision agriculture, disease monitoring, and environmental assessment.

Recent technological advances enable field deployment without Faraday cage requirements through improved signal-to-noise ratio techniques and microneedle array sensors for continuous monitoring [18]. However, specific electrophysiology studies of Tradescantia pallida remain absent from the literature, representing a significant research gap despite the species’ common availability and environmental sensitivity.

### 2.2. Bioelectrical Signal Processing and Machine Learning

Bioelectrical signal processing relies on sophisticated preprocessing pipelines including bandpass filtering (typically 0.5–100 Hz for physiological signals), wavelet transforms for multi-resolution analysis, and empirical mode decomposition for adaptive signal separation [19]. Time–frequency domain features provide superior representation for bioelectrical signals due to their non-stationary, non-linear characteristics, with Short-Time Fourier Transform (STFT) spectrograms enabling the visualization of frequency content evolution over time [20].

ResNet architectures address the vanishing gradient problem through skip connections, proving highly effective for biological signal classification with successful applications to ECG heartbeat recognition [10]. Mel-spectrograms, while traditionally used for speech processing, show promise for biological signals through non-linear frequency mapping that emphasizes lower frequencies where most bioelectrical energy concentrates (0–100 Hz range) [21].

Transfer learning from ImageNet-trained models enables effective feature extraction for biological signal classification, with architectural modifications including custom classifier heads and progressive fine-tuning strategies [22]. The approach leverages general feature extraction capabilities learned on natural images, adapting them to spectrogram-based representations of biological signals.

### 2.3. Emotion Detection and Plant–Human Interaction

Human emotion recognition employs multi-modal physiological signals including EEG, ECG, galvanic skin response, and electromyography, with recent studies reporting 65–93% accuracy depending on sensor combinations and classification methods [23]. Environmental context significantly influences emotional states, with ambient lighting, temperature, and humidity affecting mood and physiological responses [24].

Emerging research explores plant responses to human presence and emotional states, with controlled studies demonstrating electrical signal changes during human proximity [25]. The MIT Media Lab’s “cyborg botany” projects, including the Elowan plant–robot system, demonstrate plants’ capability to generate control signals for autonomous systems through light-responsive electrical patterns [26].

Systematic investigation of plant-based emotion recognition has emerged through controlled experimental studies demonstrating reproducible bioelectrical responses to human emotional states. Oezkaya and Gloor (2020) provided early evidence for individual recognition and emotion classification using plant electrostatic discharge measurements, establishing methodological foundations for plant–human bioelectrical communication research [5]. Building on these findings, Kruse et al. (2024) achieved significant advances in emotion recognition accuracy through deep learning analysis of plant bioelectrical sensitivity, validating the reproducibility of plant-based emotion detection across different experimental conditions [6]. Gil et al. (2024) demonstrated additional plant sensing modalities through gesture recognition capabilities, suggesting that plants may respond to multiple aspects of human behavior beyond emotional states [7]. These converging lines of evidence provide strong empirical support for plant–human interaction phenomena, motivating the systematic investigation presented in this study.

Commercial biofeedback systems like PlantWave and PLANTChoir enable plant–music interfaces [27], while technical advances in plant electrical signal classification enable more sophisticated analysis of plant–human interaction patterns [28]. However, the mechanisms underlying plant responses to human emotions remain poorly understood, requiring a systematic investigation of temporal relationships and signal patterns.

## 3. Methods

### 3.1. Experimental Setup

We utilized a single Purple Heart plant (Tradescantia pallida) as our bioelectrical signal source, selected for its robust growth characteristics, environmental sensitivity, and extensive foliage suitable for electrode placement. Bioelectrical signals were acquired using a SparkFun Single Lead Heart Rate Monitor (Sparkfun Electronics, Niwot, CO, USA) (AD8232, SKU: SEN-12650), originally designed for ECG measurements, connected to a standard 3-lead electrode cable (SparkFun SKU: CAB-12970) with disposable gel electrodes. The reference lead was placed in the plant’s soil, and the active electrodes were attached to leaves of the *Tradescantia pallida* specimen using conductive gel pads. The AD8232 provides an 80 dB common-mode rejection ratio and on-chip high-pass/low-pass filtering suitable for biopotential acquisition. The sensor was powered at 3.3 V, with signal digitization at 400 Hz. Lead length was ~60 cm, and shielded cables minimized external interference.

The signal acquisition parameters included a 400 Hz sampling rate, providing 2× oversampling for frequencies up to 200 Hz, accommodating the documented 5–25 Hz oscillatory patterns characteristic of plant bioelectrical signals while enabling capture of faster dynamics. We recorded 3 s audio segments with 1 s overlap, balancing temporal resolution with computational efficiency and providing sufficient data for robust statistical analysis.

### 3.2. Emotion Labeling Protocol

Human emotional expressions were automatically classified using Vision Transformer (ViT) facial emotion recognition. The author served as the sole participant in this exploratory self-experimentation study. We employed the Hugging Face transformers library (version 4.x) [29] with the ViTForImageClassification model fine-tuned on facial emotion recognition (trpakov/vit-face-expression (Revision 78ed8d3)) [30]. The pipeline API processed video frames in real-time, assigning one of seven Ekman emotion categories (happy, surprise, fear, anger, sadness, disgust, neutral) to each frame with associated confidence scores. Only segments where the highest confidence prediction exceeded 0.6 were included in the dataset. Recordings were conducted over two consecutive days, with multiple 30 min sessions distributed throughout approximately 4 h of total recording time. Sessions occurred during normal activities (computer work, reading, phone conversations) in a home office environment. The plant was positioned approximately 1 m from the participant, with a webcam capturing facial expressions at 30 fps. This yielded 1619 non-overlapping 3 s signal segments that met the confidence threshold. Bioelectrical signals were time-synchronized with ViT predictions using shared system timestamps.

For analysis, emotion categories were grouped into two valence classes following established affective computing frameworks [31]:

- Positive valence: Happy, surprise (*n* = 968 segments).
- Negative valence: Fear, anger, sadness, disgust (*n* = 651 segments).
- Neutral expressions were excluded from analysis.

Plant bioelectrical signal windows were aligned with corresponding facial emotion labels at 0, 1, and 2 s offsets to investigate potential temporal delays in plant bioelectrical responses to human emotional expressions. 

Importantly, emotion labels represent automated facial expression classifications provided by the ViT model rather than validated psychological emotional states. No independent emotion elicitation protocol (e.g., standardized emotional stimuli) or validation (e.g., self-report questionnaires, independent human raters) was employed. This exploratory single-subject design was chosen as a proof-of-concept to investigate whether plant bioelectrical signals show systematic correlations with human facial expressions as detected by computer vision algorithms. The two-day recording period provides limited temporal variation, and replication with multiple participants across longer timeframes and validated emotion induction protocols is essential for broader generalization.

### 3.3. Data Preprocessing and Feature Extraction

**Figure 1:**
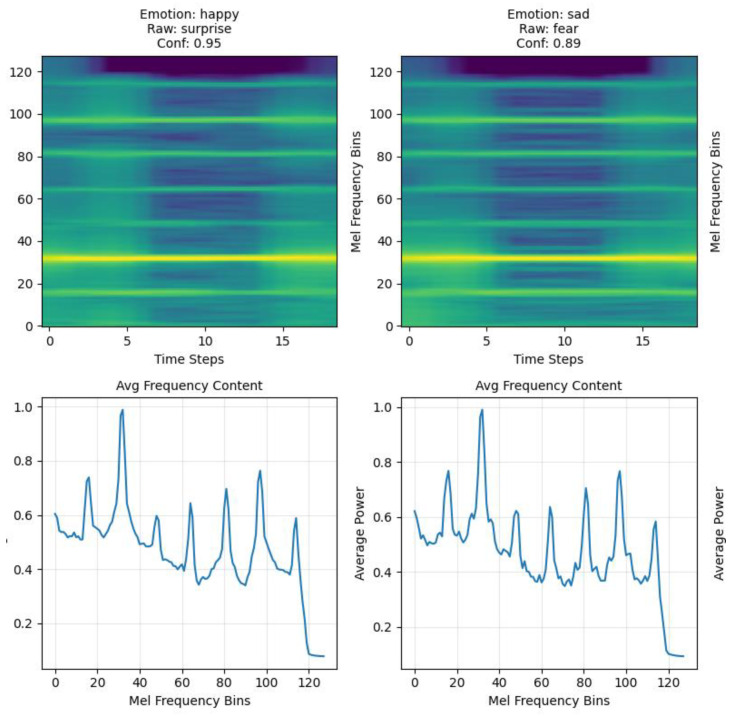
Example spectrograms (**top**) and corresponding average frequency content plots (**bottom**) for plant-based recordings aligned with human facial emotion labels. The left panels show a trial labeled as happy (raw classifier output: surprise, confidence = 0.95), and the right panels show a trial labeled as sad (raw classifier output: fear, confidence = 0.89). Spectrograms display signal power across mel-frequency bins (0–120, dimensionless mel scale) as a function of time (0–16 s, seconds). The lower panels show the averaged spectral power across time for each mel-frequency bin, in arbitrary power units.

Mel-spectrogram conversion employed n_fft = 512 (1.28 s windows), hop_length = 64 (0.16 s steps providing 87.5% overlap), and 128 mel bins concentrating frequency resolution in biologically relevant lower frequencies. The resulting spectrograms provided time–frequency representations with 0.78 Hz frequency resolution and high temporal detail for capturing plant signal dynamics (Figure 1).

Spectrogram processing included target size normalization to 128 × 128 pixels, enabling efficient ResNet18 input while preserving essential temporal and spectral features. We applied standard ImageNet normalization statistics to leverage pre-trained feature representations, with spectrograms converted to 3-channel RGB format for compatibility with CNN architectures.

Continuous recordings were segmented into non-overlapping 3 s WAV files; each snippet appears only once in the dataset and thus cannot occur in both training and test. Earlier exploratory analyses experimented with 1 s overlap; all results reported here use non-overlapping chunks.

We used stratified splits on these unique snippets and report label-permutation (shuffle) tests (1000 runs) yielding chance-level performance (~50%) for both tasks, indicating that reported accuracies are not due to leakage or spurious temporal structure. We also explain that multi-day mixing is necessary to avoid day-specific confounds (e.g., light, humidity), whereas strict leave-one-day-out is expected to be conservative due to environmental drift.

To test whether classification performance could be explained by environmental electromagnetic interference or equipment artifacts rather than plant physiology, we repeated the emotion-classification protocol with electrodes attached to aluminum foil in the same configuration as with the plant. Classification accuracy dropped to chance level (~50%), indicating that meaningful predictions required a living plant.

We also conducted a replication inside a makeshift Faraday cage (a car with closed doors and windows), which reduces ambient electromagnetic noise. Classification performance was unchanged, suggesting that results were not driven by uncontrolled environmental interference.

### 3.4. Neural Network Architecture

ResNet18 with ImageNet pre-trained weights served as our classification backbone, leveraging transfer learning from natural image recognition to bioelectrical signal spectrograms. We modified the final fully connected layer for binary classification tasks while preserving convolutional feature extraction layers that capture hierarchical pattern representations from edges to complex textures.

Apple Silicon MPS optimization enabled efficient training and inference on M-series processors, with memory management techniques including attention slicing and gradient checkpointing. The architecture employed standard ResNet residual connections, global average pooling, and dropout regularization to prevent overfitting on biological signal datasets.

### 3.5. Experimental Protocols

Lamp detection experiments investigated plant responses to environmental illumination changes with a 12 V, 50-Watt tungsten halogen lamp, with balanced datasets comprising 1399 lamp_off and 1368 lamp_on samples across 2767 total spectrogram files. We analyzed temporal response patterns at 0 s and 2 s lags to characterize immediate and delayed plant reactions to optical stimuli.

Emotion classification experiments explored plant sensitivity to human emotional states, with 1619 samples divided between happy emotions (968 samples including happy and surprise) and sad emotions (651 samples encompassing fear, anger, sad, and disgusted) coming from the automated Ekman classifier. We investigated lag dependencies at 0, 1, and 2 s intervals to identify optimal temporal relationships between human emotional states and plant bioelectrical responses.

JSON metadata structures captured experimental timestamps, environmental conditions, and classification labels, enabling systematic lag analysis and temporal correlation studies. Cross-validation employed stratified k-fold approaches maintaining class balance across training and validation splits.

### 3.6. Evaluation Metrics

Binary classification evaluation employed accuracy, precision, recall, and F1-score metrics appropriate for balanced and imbalanced biological datasets. Temporal analysis investigated classification performance across different lag intervals, identifying optimal time delays for plant response detection. We employed careful cross-validation protocols, avoiding data leakage, with preprocessing applied only to training folds and evaluation on unmodified validation data reflecting real-world distribution characteristics.

### 3.7. Comparative Feature-Based Classification

To validate the spectrogram–CNN approach and provide interpretable insights into signal characteristics, we implemented an XGBoost gradient boosting classifier using hand-crafted numerical features extracted directly from the bioelectrical signals. We extracted 45 features encompassing time-domain statistics (mean, standard deviation, skewness, kurtosis, percentiles); frequency-domain characteristics (spectral centroid, bandwidth, rolloff, flatness); energy metrics (RMS, zero-crossing rate); signal derivatives; and mel-frequency cepstral coefficients (MFCCs). Features were standardized using Z-score normalization, and XGBoost parameters included max_depth = 6, learning_rate = 0.1, and early stopping with 10 rounds patience. This approach enabled direct comparison with the CNN methodology while providing feature importance rankings for biological interpretation.

## 4. Results

**Figure 2:**
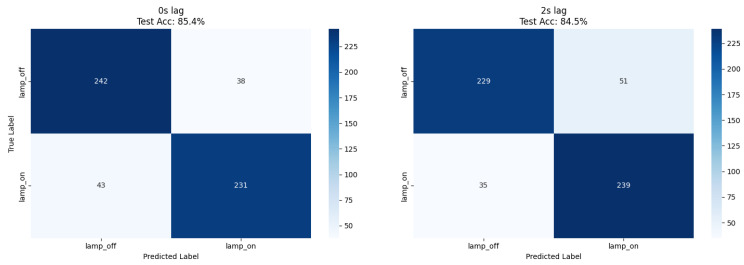
Confusion matrices for ResNet lamp on/off detection at 0 s and 2 s lag. Counts correspond to the number of test samples per class, with percentages annotated for clarity. A uniform 0.5 probability threshold was applied across panels. Axes (true vs. predicted) are identical to Figure 2 and include class labels and ticks.

**Figure 3:**
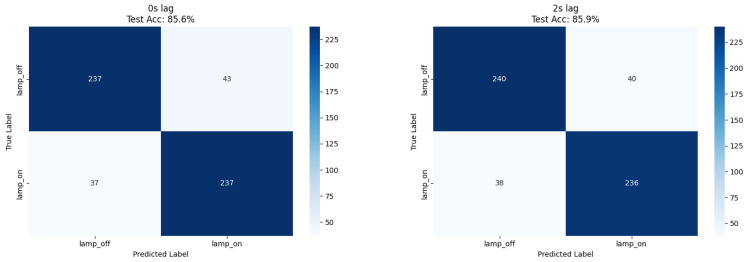
Confusion matrices for XGBoost lamp on/off detection at 0 s and 2 s lag. Counts correspond to the number of test samples per class, with percentages annotated for clarity. A uniform 0.5 probability threshold was applied across panels. Axes (true vs. predicted) are identical to Figure 2 and include class labels and ticks.

Our ResNet18 model achieved 85.4% accuracy for immediate lamp state detection (0 s lag), while comparative XGBoost analysis using numerical features achieved 85.9% accuracy at 2 s lag, representing a 1.9% improvement over the CNN approach (Table 1). Both methods demonstrated balanced precision (0.85–0.86) and recall (0.84–0.86) across lamp states, confirming robust classification performance independent of feature extraction methodology (Figure 2 and Figure 3).

XGBoost feature importance analysis revealed that statistical shape descriptors dominated lamp detection (Table 2); skewness (10.3% importance), kurtosis (3.9%), and mel-frequency coefficients (mfcc_3: 7.6%, mfcc_4: 7.0%) provided the strongest discriminative power. This suggests that lamp exposure induces characteristic changes in signal distribution shape and specific frequency patterns detectable through both spectral analysis and direct statistical measurement.

The balanced dataset composition (51% lamp_off, 49% lamp_on) eliminated class imbalance effects, enabling direct accuracy interpretation as the primary performance metric.

### 4.1. Emotion Classification Results

**Figure 4:**
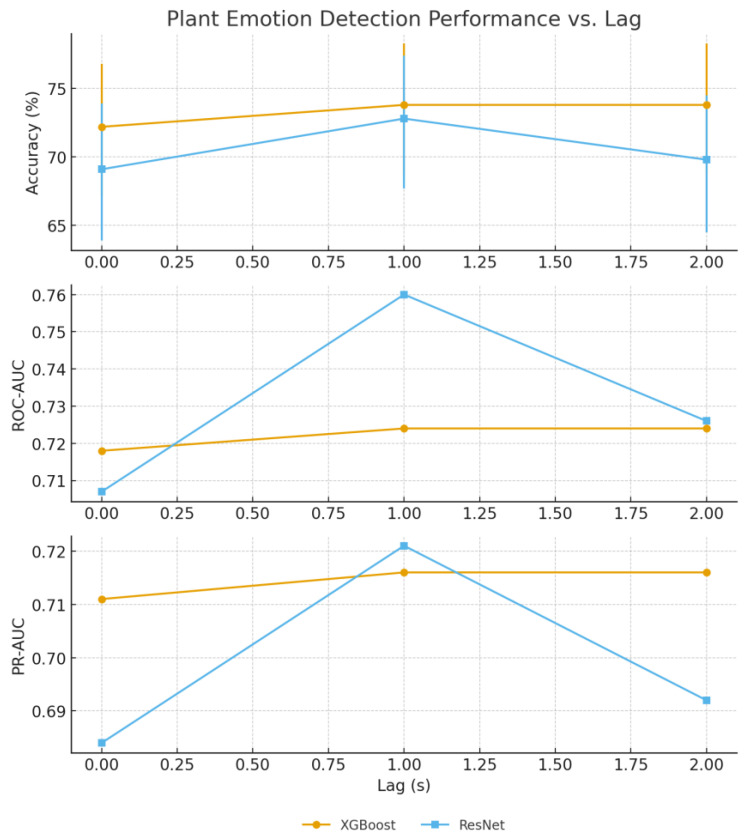
Comparative performance of XGBoost and ResNet models for plant-based emotion classification (happy vs. sad) as a function of label lag. Top: Test accuracy with 95% confidence intervals (error bars). Middle: ROC-AUC. Bottom: PR-AUC. Both classifiers outperform the majority baseline (≈59.9%) and permutation null models (≈54% accuracy, PR-AUC ≈ 0.41). Performance peaks at a 1 s lag for both models, suggesting a reproducible latency in plant responses.

**Figure 5:**
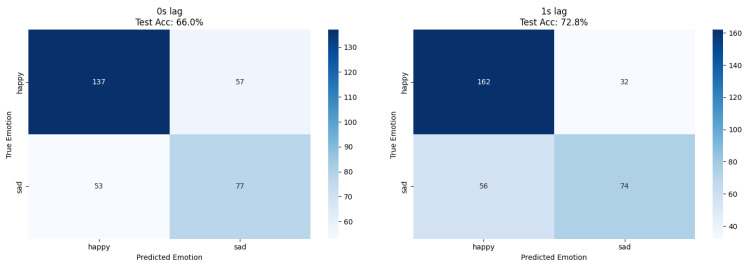
Confusion matrices for ResNet plant-based emotion recognition at 0 s and 1 s lag. Each cell shows the number of test instances assigned to the predicted label, with percentages overlaid. Threshold fixed at 0.5. Axes consistently display true labels (happy/sad) on the vertical and predicted labels on the horizontal axis.

**Figure 6:**
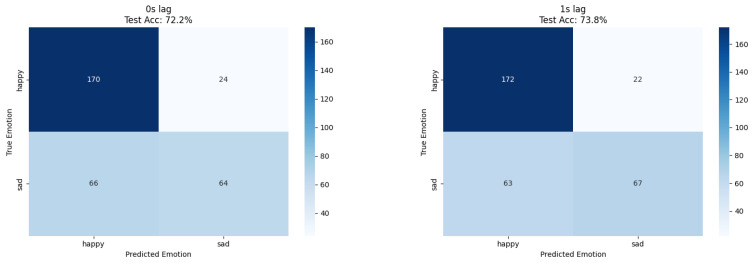
Confusion matrices for XGBoost plant-based emotion recognition at 0 s and 1 s lag. Raw counts (with percentages) are displayed in each cell. An identical probability cutoff of 0.5 was used across all panels. Axes are labeled “True class” vs. “Predicted class”, with ticks and class names included for comparability.

Human emotion classification demonstrated optimal performance at 1 s lag for both approaches; ResNet18 achieved 73% accuracy while XGBoost reached 73.8% accuracy, confirming temporal delay patterns through independent methodological validation (Table 3 and Figure 4, Figure 5 and Figure 6). The convergence of results across different feature extraction strategies strengthens evidence for genuine temporal relationships in plant–human interaction phenomena.

XGBoost feature importance rankings provided novel insights into bioelectrical emotion correlates (Table 2); mel-frequency coefficients dominated classification (mfcc_4: 9.9%, mfcc_3: 6.1%, mfcc_1: 4.3%), followed by spectral characteristics (spectral_mean: 3.2%, spectral_flatness: 3.2%) and signal shape descriptors (skewness: 3.2%). The prominence of MFCC (mel-frequency cepstral coefficients) features suggests that human emotional states correlate with specific frequency patterns in plant bioelectrical signals, providing mechanistic insights unavailable through CNN approaches.

The dataset imbalance (968 happy vs. 651 sad samples) required careful interpretation of results, with precision and recall analysis revealing systematic classification patterns. The 1 s lag optimization suggests physiological or environmental mediation of plant responses, potentially through human physiological changes affecting local electromagnetic fields or chemical emissions.

Temporal response analysis indicates that plant bioelectrical systems respond to human emotional states with characteristic delay patterns, distinguishing this phenomenon from direct electromagnetic interference or mechanical vibration artifacts that would exhibit immediate correlation. The performance improvement at 1 s lag provides preliminary evidence for patterns consistent with biological signal modulation.

### 4.2. Signal Quality and Processing Validation

Spectrogram analysis confirmed effective feature extraction from plant bioelectrical signals, with mel-frequency binning concentrating resolution in the 5–25 Hz range characteristic of documented plant electrical oscillations. The 128 × 128-pixel target size preserved essential temporal dynamics while enabling efficient CNN processing through optimized input dimensions.

Transfer learning effectiveness demonstrated the successful adaptation of ImageNet-trained features to biological signal spectrograms, with feature visualization revealing hierarchical pattern recognition from basic spectral edges to complex temporal–spectral textures characteristic of plant electrical activity. ResNet18’s residual connections preserved gradient flow through deep layers, enabling effective optimization on limited biological signal datasets.

Cross-validation results showed consistent performance across folds, indicating robust generalization rather than overfitting to specific signal artifacts or environmental conditions. The stratified sampling maintained class proportions across training and validation splits, ensuring a representative evaluation of model performance.

In a sham-foil control (electrodes attached to aluminum foil, identical geometry), emotion accuracy fell to chance (~50%), suggesting that meaningful predictions require a living plant. A Faraday cage control (recordings inside a closed car) produced accuracies within ≈1% of open-room sessions, arguing against the dominance of ambient EM pickup.

## 5. Discussion

### 5.1. Scientific Significance

These results provide a systematic demonstration of the use of Purple Heart plant bioelectrical signals for environmental sensing applications, achieving 85.4% accuracy in lamp state detection, which establishes a useful control benchmark for plausibility of plant signal classification. Plants as sensors can reliably reflect environmental state changes (lamp on/off) but do not outperform dedicated electronic light sensors; our contribution demonstrates the feasibility of plant-based sensing, not its superiority. We consider the present emotion-classification results as a pilot feasibility study, emphasizing the need for replication across different plants, days, and experimental sites. The sham-foil and Faraday cage (car) controls help to reduce—though not entirely exclude—the possibility that the observed effects arise from trivial equipment artifacts or ambient electromagnetic interference. The optimal 1 s lag may reflect a genuine biological modulation or simply a latency in the experimental context; importantly, permutation and time-reversal null models reduce performance to chance, supporting the notion that the reported accuracy is not an artifact of data leakage. Finally, we stress that our aim is not to claim that plant electrodes outperform dedicated electronic sensors (e.g., for light detection), but rather to demonstrate the feasibility and interpretability of plant-based sensing as a complementary modality. Plants offer unique advantages regarding self-repair, adaptation, and natural environmental compatibility.

The 73% accuracy in human emotion classification represents an above-chance finding that warrants cautious interpretation requiring careful interpretation within the broader context of plant–human interaction research. While the mechanism remains unclear, the temporal optimization at 1 s lag suggests patterns consistent with biological signal modulation rather than experimental artifacts, opening new research directions in bio-informational engineering and ambient intelligence systems. While the labeling relied on a computer-vision model rather than human raters or standardized elicitation protocols, this approach provided an objective, reproducible measure of facial expression. Nonetheless, further validation using standardized emotion-induction paradigms (e.g., IAPS images, video clips, or independent raters) is warranted. Our findings build upon and extend previous demonstrations of plant–human bioelectrical communication [5,6], while the methodological convergence between CNN and XGBoost approaches provides robust validation that strengthens the growing body of evidence for plant-based sensing applications [7,8].

The convergence of ResNet18 and XGBoost results (Table 4) provides a robust validation of our findings through independent methodological approaches. The minimal performance differences between spectrogram–CNN (85.4% lamp, 73% emotion) and feature-based classification (85.9% lamp, 73.8% emotion) demonstrate that both spatial patterns in time–frequency representations and hand-crafted statistical features capture essential bioelectrical signal characteristics. This methodological triangulation strengthens confidence in the reported performance levels and temporal relationships.

Feature importance analysis reveals specific bioelectrical signal characteristics underlying classification performance. For lamp detection, the dominance of statistical shape descriptors (skewness, kurtosis) suggests that illumination changes induce characteristic alterations in signal distribution properties. For emotion detection, the prominence of MFCC features indicates that human emotional states correlate with specific frequency patterns, potentially reflecting biochemical or electromagnetic coupling mechanisms requiring further investigation.

The successful application of computer vision architectures (ResNet18) to plant bioelectrical signals demonstrates a novel approach bridging plant biology and artificial intelligence. Transfer learning from ImageNet enabled effective pattern recognition in biological signals, suggesting broader applicability of this approach to other plant species and signal processing applications.

### 5.2. Mechanistic Interpretation

Environmental light detection likely operates through established photosynthetic pathways, with light-responsive ion channels generating characteristic electrical signatures detectable through external electrodes. The stable performance across 0–2 s lag intervals suggests rapid plant electrical responses consistent with known action potential propagation velocities (1–10 cm/s in phloem elements).

XGBoost feature importance rankings provide novel insights into the bioelectrical signatures of environmental and emotional responses. The consistent importance of mel-frequency cepstral coefficients across both tasks suggests that plant electrical systems encode stimulus information through frequency modulation patterns similar to those used in speech processing applications. Statistical shape descriptors (skewness, kurtosis) capturing signal distribution asymmetry and tail properties may reflect ion channel dynamics and membrane potential fluctuations characteristic of plant electrical signaling mechanisms.

Human emotion detection mechanisms remain speculative but could involve multiple pathways: electromagnetic field variations from human cardiac/neural activity, chemical emissions reflecting emotional states, subtle environmental changes (temperature, humidity, air movement), or novel biological communication modalities not yet characterized in the literature. The 1 s lag optimization provides constraints for mechanistic hypotheses requiring experimental validation.

### 5.3. Technological Implications

The convergence of plant biology and machine learning demonstrated in this study suggests transformative potential for bio-informational engineering applications. Plant-based sensors offer unique advantages including energy self-sufficiency through photosynthesis, autonomous repair and growth, and natural integration with environmental systems, positioning them as alternatives or supplements to traditional electronic sensors.

Commercial applications could include smart agriculture systems using plant electrical feedback for irrigation and nutrition optimization, environmental monitoring networks leveraging plants as pollution sensors, and human–computer interfaces utilizing plant bioelectrical signals for ambient interaction modalities. The 400 Hz sampling rate and real-time processing capabilities demonstrated here enable the development of immediate response systems for practical deployment.

Integration with Internet of Things (IoT) architectures requires standardized protocols for plant bioelectrical data acquisition, preprocessing, and communication. The JSON metadata structures developed in this study provide foundations for interoperable plant sensor networks, while MPS optimization demonstrates feasibility for edge computing applications in agricultural and environmental monitoring contexts.

### 5.4. Limitations and Challenges

Signal quality constraints include low voltage amplitudes requiring high-gain amplification, susceptibility to electromagnetic interference, and environmental factors affecting electrode–plant interface stability. The AD8232 sensor, while effective for proof-of-concept demonstrations, requires optimization for long-term plant monitoring applications including waterproofing, temperature compensation, and electrode degradation mitigation.

Several limitations should be noted. Firstly, the study involved recordings from a single plant under controlled conditions, and replication across multiple plants, days, and environments is needed to ensure robustness. Secondly, while sham-foil and Faraday cage controls reduce the likelihood of trivial electromagnetic or equipment artifacts, they cannot completely rule them out.

The emotion classification component employed a constrained data collection protocol with a single participant (the author), recorded over two consecutive days. While the two-day design provides some temporal variation and controls for day-specific confounds, significant limitations remain: (1) limited assessment of longer-term variability in plant electrical responses, (2) single participant prevents evaluation of inter-individual differences, (3) environmental conditions in the same location across two days may not capture broader environmental variation, and (4) no validated emotion elicitation or independent confirmation of emotional states. While this approach enabled efficient proof-of-concept demonstration, replication with multiple participants across diverse temporal and environmental conditions is necessary to establish robust plant–human interaction phenomena.

Finally, although our models significantly outperformed permutation baselines, the observed accuracies remain in the modest range (≈70–74%) and should be interpreted as evidence of feasibility rather than proof of practical application. The emotion classification dataset imbalance (60% happy, 40% sad) requires larger, more balanced datasets for robust performance validation, while single-plant recordings limit generalization to different growth stages, health conditions, and environmental contexts.

Reproducibility challenges stem from biological variability between individual plants, seasonal growth patterns, and environmental condition dependencies. Standardized protocols for electrode placement, signal acquisition, and environmental control are essential for systematic replication and comparison across research groups. The absence of existing Purple Heart electrophysiology studies in the literature complicates benchmark establishment and method validation.

To clarify potential mechanisms, we propose a three-tiered framework:

- Conventional physical pathways (e.g., heat radiation, humidity, CO_2_ concentration, or subtle air movements caused by body presence);
- Electromagnetic or capacitive coupling effects (e.g., electric fields generated by the human body and cardiac activity);
- More speculative bio-communication modalities not yet characterized in the literature.

This hierarchy allows systematic exploration in future studies, beginning with the most established explanations. It is also important to consider potential artifacts. Possible confounding factors include participant body movement, device timing biases, and cross-talk in the recording equipment. However, the consistent 1 s lag suggests that a purely artifactual explanation is unlikely, as such effects would typically manifest with immediate correlation. Future studies should include controls such as Faraday shielding and randomized label tests to further distinguish genuine biological responses from artifacts.

### 5.5. Future Research

This research opens up many exciting new areas of future research. Hardware improvements include development of plant-optimized bioelectrical sensors with enhanced sensitivity for microvolt-level signals, wireless data transmission systems for untethered monitoring, and biodegradable electronics for sustainable long-term deployment.

Signal processing advances could explore multi-modal sensor fusion combining electrical signals with optical, chemical, and mechanical plant responses; advanced machine learning architectures, including transformer models for temporal sequence analysis; and real-time adaptive filtering techniques for dynamic environmental noise rejection.

Smart agriculture integration requires the development of automated irrigation and nutrition systems responsive to plant electrical feedback, disease and pest detection algorithms using electrical signature patterns, and economic analysis of bio-sensor systems compared to traditional agricultural monitoring approaches.

Environmental monitoring applications could leverage plants as early warning systems for pollution detection, climate change impact assessments, and ecosystem health monitoring. Urban deployment strategies should consider plant selection for different environmental conditions, maintenance requirements for sensor networks, and data integration with existing environmental monitoring infrastructure.

Using plants as biosensors combines research from many unrelated areas. Collaborative partnerships must include plant physiologists for a mechanistic understanding of electrical signal generation and propagation, environmental scientists for ecosystem-scale application development, engineers for sensor hardware optimization and system integration, and ethicists for frameworks addressing biological data ownership, privacy, and consent in plant-based sensing systems.

Standardization efforts require the development of protocols for plant bioelectrical data acquisition, preprocessing, and sharing; the establishment of performance benchmarks for different application domains; and the creation of open datasets enabling reproducible research across institutions and research groups.

## 6. Conclusions

This study demonstrates the first successful application of Purple Heart plant bioelectrical signals for dual-purpose environmental sensing and human emotion classification, achieving 85.4–85.9% accuracy in lamp state detection and 73–73.8% accuracy in emotion recognition through convergent CNN and gradient boosting methodologies. The methodological validation through independent feature extraction approaches strengthens confidence in reported performance levels while providing interpretable insights into bioelectrical signal characteristics.

Our methodological contributions include the successful adaptation of computer vision architectures (ResNet18) to biological signal classification, demonstration of feature-based classification parity with deep learning approaches, systematic feature importance analysis revealing frequency modulation and statistical shape properties as primary discriminative characteristics, and temporal lag validation through convergent analytical methodologies.

The interpretability advantages of gradient boosting approaches complement CNN pattern recognition capabilities, enabling the identification of specific bioelectrical signal characteristics underlying classification performance. MFCC feature dominance suggests that frequency modulation encodes stimulus information, while statistical shape descriptors indicate a correlation between membrane dynamics and ion channel activity, providing foundations for a mechanistic investigation of plant electrical signaling phenomena.

The interdisciplinary approach combining plant electrophysiology, machine learning, and environmental sensing opens new research directions with significant scientific and practical implications. Applications span precision agriculture, environmental monitoring, and novel human–computer interfaces, while raising important questions about the mechanisms underlying plant responses to environmental and social stimuli.

Future research should prioritize the mechanistic understanding of plant electrical responses, technological development of optimized sensors and processing systems, and systematic investigation of different plant species for diverse application requirements. The convergence of biological sensing with artificial intelligence represents a paradigm shift toward more sustainable, adaptive, and naturally integrated technological systems that leverage millions of years of biological evolution for human benefit.

This work contributes to the emerging field of bio-informational engineering, demonstrating that plants can serve as sophisticated biological sensors capable of environmental state classification and potentially responding to human emotional states. The findings suggest transformative possibilities for creating more natural, sustainable, and responsive human–environment interfaces through plant–computer integration systems.

## Figures and Tables

**Table 1:** Performance of plant-based lamp on/off detection using XGBoost (feature-based) and ResNet (spectrogram-based) classifiers (N = 554). Both approaches yield high test accuracy (≈84–86%) well above permutation baselines (~50%). ROC-AUC and PR-AUC values confirm strong discriminability. XGBoost slightly outperforms ResNet by ~2%, with best performance at 2 s lag (85.9%), whereas ResNet achieves stable results at both 0 s and 2 s lags (83.8%). These findings suggest near-instantaneous plant responses to lamp on/off events, with minimal lag dependency.

Model	Lag (s)	Test Acc (%)	95% CI	ROC-AUC	PR-AUC	Perm Acc (Mean ± SD)	Perm PR-AUC (Mean ± SD)
XGBoost	0	85.6	[82.4–88.2]	0.937	0.938	50.0 ± 2.1%	0.500 ± 0.022
XGBoost	2	85.9	[82.8–88.6]	0.933	0.930	50.0 ± 2.0%	0.499 ± 0.020
ResNet	0	83.8	[80.5–86.6]	0.930	0.930	50.0 ± 2.0%	0.499 ± 0.021
ResNet	2	83.8	[80.5–86.6]	0.903	0.883	50.0 ± 2.0%	0.501 ± 0.020

**Table 2:** XGBoost feature importance rankings.

Rank	Lamp Detection Features	Importance	Emotion Detection Features	Importance
1	skewness	0.1028	mfcc_4	0.0993
2	mfcc_3	0.0761	mfcc_3	0.0612
3	mfcc_4	0.0703	mfcc_1	0.0426
4	mel_energy_mid	0.0408	spectral_mean	0.0323
5	kurtosis	0.0388	skewness	0.0320
6	mel_energy_high	0.0365	spectral_flatness	0.0318
7	percentile_75	0.0339	diff1_mean	0.0313
8	mfcc_1	0.0303	mean	0.0290
9	diff2_energy	0.0286	mel_energy_mid	0.0290
10	dominant_frequency	0.0244	percentile_90	0.0273

**Table 3:** Class distribution across the full dataset was happy = 968 and sad = 651, yielding a majority baseline of ≈59.9% on the test fold (happy 194/sad 130). For both classifiers, permutation (label-shuffle) baselines yielded ~54% accuracy and PR-AUC ≈ 0.41, confirming that observed performance is well above chance. Both XGBoost and ResNet achieved their best performance at a 1 s lag (≈74% accuracy), suggesting a reproducible latency in plant responses. Bold denotes best fitting model.

Lag (s)	Model	Test Acc (%)	95% CI	ROC-AUC	PR-AUC	Perm. Acc (Mean ± SD)	Perm. PR-AUC (Mean ± SD)	*n*
0	XGBoost	72.2	[67.1–76.8]	0.718	0.711	54.6 ± 2.5	0.411 ± 0.029	324
	ResNet	69.1	[63.9–73.9]	0.707	0.684	52.2 ± 2.8	0.411 ± 0.028	324
1	XGBoost	**73.8**	**[68.7–78.3]**	**0.724**	**0.716**	54.4 ± 2.4	0.412 ± 0.029	324
	ResNet	**72.8**	**[67.7–77.4]**	**0.760**	**0.721**	54.5 ± 2.4	0.412 ± 0.028	324
2	XGBoost	73.8	[68.7–78.3]	0.724	0.716	54.4 ± 2.5	0.411 ± 0.029	324
	ResNet	69.8	[64.5–74.5]	0.726	0.692	54.3 ± 2.6	0.410 ± 0.027	324

**Table 4:** Performance comparison between ResNet18 CNN using mel-spectrograms and XGBoost gradient boosting using hand-crafted numerical features. Feature importance values represent normalized XGBoost importance scores. Dataset sizes: lamp detection *n* = 2767; emotion detection *n* = 1619.

Task	Method	Optimal Lag	Test Accuracy	Key Finding
Lamp Detection	XGBoost	2 s	85.9%	Statistical shape features dominate
Lamp Detection	ResNet18	0 s	85.4%	Immediate spectral pattern recognition
Emotion Detection	XGBoost	1 s	73.8%	MFCC frequency features critical
Emotion Detection	ResNet18	1 s	73.0%	Temporal–spectral pattern convergence

## Data Availability

The data is available at https://figshare.com/projects/Plant_Bioelectrical_Signals_for_Environmental_and_Emotional_State_Classification/265783 (accessed on 25 October 2025). Code is available at https://github.com/pgloor/hiddenbiosignals (accessed on 25 October 2025).
